# Estimating the economic burden of COVID-19 survivors in Punjab, Pakistan

**DOI:** 10.1371/journal.pone.0337995

**Published:** 2026-01-05

**Authors:** Shumaila Abbas, Jamal Abdul Nasir, Rehan Ahmad Khan Sherwani

**Affiliations:** 1 Department of Statistics, Government College University, Lahore, Pakistan; 2 College of Statistical Sciences, University of the Punjab, Lahore, Pakistan; STIKES Wira Medika PPNI Bali: Sekolah Tinggi Ilmu Kesehatan Wira Medika PPNI Bali, INDONESIA

## Abstract

**Background:**

The COVID-19 survivors are under a great deal of financial stress due to high medical costs, income lost during illness, and ongoing medical expenses. Many survivors borrow money, deplete their savings, or become more economically vulnerable. The situation becomes worse for the marginalized groups who do not receive enough support.

**Aims and objectives:**

The pandemic has also had a significant financial and social impact on survivors in Pakistan. The present research aims to quantify the economic implications of COVID-19 on survivors from Punjab, Pakistan.

**Methods:**

A cross-sectional study of 5045 survivors from Punjab, Pakistan, was conducted, and the economic burden of COVID-19 on survivors was quantified using a 27-item self-administered scale. Descriptive statistics, structural equation models, and odds ratios were computed to quantify the study’s objectives.

**Results:**

The economic burden was classified into six constructs, representing the financial and social burdens of the disease on survivors. About 59.2% of the survivors were male, with an average age of 45.4 years, and 65.8% were employed in some capacity. The results depicted that the survivors aged over 45 years (OR=2.15, p < 0.01), admitted to hospital (OR=2.32, p < 0.01), infected severely (OR=2.11, p < 0.01), maximum secondary level of education (OR=2.18, p < 0.01), and disease duration up to 14 days (OR=5.05, p < 0.01) have an elevated financial burden than the opponent groups. The average duration of the disease was 12.1 days, and the daily cost of living and morbidity was estimated at 4763 PKR and 21286 PKR, respectively.

**Conclusion:**

The financial burden of the disease not only affects survivors but also strains families and communities, underscoring the necessity of comprehensive support systems and policies to address the social and economic impacts of the pandemic on survivors. Policymakers should target healthcare cost containment, income support, and financial support to vulnerable groups such as women, unemployed individuals, and those infected with severe diseases.

## 1. Introduction

The COVID-19 pandemic has had a significant impact on the global economy, particularly in Asia and the Pacific, including Pakistan [1]. Extended health concerns, such as social, physical, and psychological, repeatedly entail enduring healthcare, leading to elevated out-of-pocket expenditures, particularly in low-income countries. Multiple research studies from different countries have indicated that survivors often spend their savings, incur debt, or borrow to pay for medications, thereby increasing their economic risk [2,3]. The global economic burden of COVID-19 has been substantial, with estimates suggesting a total direct medical cost of $163.4 billion in the United States throughout the pandemic, based on a 20% infection rate [4]. The economic burden of COVID-19 has been multifaceted, affecting various sectors, including healthcare, tourism, trade, and the environment. A study by [5] emphasized considerable economic stress on healthcare structures due to diagnosis, treatment, and hospitalization expenses while estimating the financial burden of disease worldwide. The financial burden worsens due to indirect costs, including supply chain disruptions and production losses [6–8]. Long-term impacts, such as government stimulus measures and changes in consumer behavior, also contributed to the financial decay of COVID-19 [9]. The COVID-19 pandemic had caused both demand and supply shocks to reverberate through the global economy, affecting over 210 countries with more than 67 million confirmed cases and over 1.5 million deaths worldwide, including Pakistan [10]. This economic downturn has been attributed to pandemic-related impediment measures, such as lockdowns, social distancing, and travel restrictions, which have significantly impacted the livelihoods of millions of workers, with a projected rise of 33.7% in the poverty level [11].

Nevertheless, less research had been conducted to understand the financial consequences of COVID-19 survivors in developing countries like Pakistan, where accessible healthcare and better social protection are relatively scarce. According to a study by [12], vulnerable groups in society, including low-income earners, female individuals, and unemployed populations, were among the biggest losers in terms of the economic impacts of the pandemic. Many of these groups did not receive proper healthcare and financial support to address their needs, and as a result, they faced hardships. Other studies in low- and middle-income countries observed the same pattern, where the pandemic only deepened societal inequalities [6,12,13].

Pakistan’s healthcare system was overwhelmed during the pandemic due to a scarcity of resources and a large population that needs healthcare. According to data presented by [14], Pakistan’s out-of-pocket healthcare spending was among the highest in South Asia. Pakistan, with its high population density, limited healthcare capacity, and existing poverty, faced rapid community transmission of the coronavirus. The pandemic had resulted in significant economic losses, with estimates suggesting a 10% economic contraction, equivalent to 1.1 trillion PKR, in the fiscal year 2021 [15]. The pandemic impediment measures, including lockdowns, social distancing, and travel restrictions, had substantially impacted the livelihoods of approximately 7.15 million workers, leading to a projected 33.7% rise in poverty [11]. While the economy’s primary, secondary, and tertiary sectors had been adversely affected, lockdowns had paradoxically led to a drastic improvement in the air quality index of urban centres in Pakistan [14]. Similarly, [16] also concluded that rural patients and low-education patients in Pakistan who survived COVID-19 experienced a significant economic burden because of an absence of adequate healthcare facilities and resources.

Furthermore, the pandemic disrupted global trade, decreased Pakistan’s exports and imports, and had a significant impact on its overall economy [17]. The country’s total debts and liabilities had increased significantly, indicating a challenging situation for debt servicing and perpetuating a cycle of dependency on the developed world [18]. The negative economic growth and substantial contractions in various financial parameters, such as GDP growth rate, inflation, per capita income, debt, tax collection, and trade, have further underscored the significant economic challenges faced by Pakistan. The need for research has been raised due to the limited data on the financial impacts of COVID-19 on survivors, especially in developing countries such as Pakistan, where more than 60% of the population works in the informal economy and has poor access to health insurance or paid sick leave. Although the global analysis presented the effects of the pandemic on the economic front, there were minimal reports on how survivors in Punjab, Pakistan, managed medical costs, income loss, and recurring expenses. This study fills this gap by targeting core demographical characteristics, including age, gender, employment status, and the severity of the disease, to guide policymakers in designing the right interventions for the financially struggling COVID-19 patients in Pakistan.

This research aims to quantify the financial strain of COVID-19 among survivors residing in Punjab, Pakistan. This study offers a novel examination of the economic burden on COVID-19 survivors in Punjab, Pakistan, an underexplored area in the context of post-pandemic financial recovery. Focusing on survivors and utilizing a comprehensive 27-item scale quantifies the long-term financial stress caused by COVID-19. The study provides significant findings about how the pandemic deepens economic risks and affects developing countries like Pakistan. This paper quantifies the likelihood of financial stress in these populations – women, the unemployed, and those affected by COVID-19– providing a basis for intervention. The research question stems from the idea that people who contracted COVID-19 are financially insecure because of outstanding medical expenses and loss of income, as well as limited financial support. This burden proportionately falls on the poor groups, thus making them economically vulnerable and highlighting the need for structural modifiers and support-oriented policies in Pakistan. The rationale for this research originates from the fact that COVID-19 survivors encounter severe economic impacts in low-income countries, including the context of Pakistan. It is essential to note that there is currently limited information on the financial effects experienced by these survivors; therefore, the study aims to fill this gap while emphasizing the importance of research-informed policy responses. The main objective is to evaluate how COVID-19 affects survivors regarding financial indicators and determine which groups are most at risk and the causes of their economic dependency. It aims to provide information that may help the authorities design effective actions. The general hypothesis is that COVID-19 survivors experienced more economic loss than other groups, including the elderly, women, married individuals, the unemployed, and those with lower educational levels. A higher financial burden is also observed among patients with severe disease, ICU admission, or short duration of illness.

## 2. Materials and methods

The study was approved by the IRB, Department of Statistics, GCU Lahore-Pakistan vide protocol No. St-04A/23 dated 02-01-2023. The target population of the present study was the PCR-diagnosed COVID-19 survivors in Punjab-Pakistan on May 01, 2021. The registered survivors in the province on the date was 303182, with an infection rate of 9.63%. The appropriate sample size for the finite population (N=303182) with a 95% confidence level (Z=1.96), 5% margin of error (e=0.05), and 9.63% positivity ratio (p=0.0963) were computed as [19]:


n=Z2*p^*(1−p^)e2/Z2*p^*(1−p^)e21+Z2*p^*(1−p^)e2*N\nulldelimiterspace1+Z2*p^*(1−p^)e2*N=5045

A cross-sectional study design was employed to collect the required data from the target population using a convenience sampling technique. The possible limitations of using convenience sampling include bias, unrepresentativeness, and limited generalizability. The potential limitations of using convenience sampling are mitigated to some extent by the large sample size of 5,045 survivors. A sample of this magnitude, combined with the diverse demographic representation, provides a robust dataset that captures a broad range of experiences and financial burdens across different groups. While random sampling could have further enhanced generalizability, the large and diverse sample in our study still provides valuable insights and meaningful patterns relevant to understanding the economic burden of COVID-19 survivors in Punjab. Under this scheme, various approaches were employed, including the use of a Google form, where the online form was shared with individuals via email, social groups, and other means, and they were asked to participate in the research only if they had confirmed a positive PCR test result for COVID-19. Moreover, face-to-face data was collected from the respondents interested in participating in the study. The researcher or trained enumerators contacted these respondents in public places, shopping malls, markets, home visits, institutions, offices, etc. In all these methods, formal written consent was obtained from the respondents before they participated in the research. A binary response twenty-seven items questionnaire with some demographic information was designed to measure the financial impact of COVID-19 on the survivors after an extensive literature review and focus group sessions. All items on the scale are presented as yes or no, making it easier to understand and administer. The focus group sessions contain six to ten stakeholders. After pretesting from 50 survivors, the questionnaire was modified, and the reliability [20] of the constructs is outlined in Table 1.

**Table 1:** Reliability statistics of the financial burden questionnaire and the constructs.

Construct	Items	Reliability Measure
Financial Outlay	7	0.788
Financial Hindrance	4	0.784
Hostile Behavior	4	0.767
Care and Lifestyle	3	0.779
Seeking Support	4	0.737
Psychosocial Affect	5	0.764
**Overall**	**27**	**0.916**

The Cronbach’s alpha values indicated satisfactory results for the scale development. The questionnaire was designed to assess the financial burden on six constructs experienced by COVID-19 survivors. These constructs include:

**Financial outlay (FO):** The construct FO with seven items covers the survivors’ cut on the routine budget due to COVID-19. These cuts include spending on food and recreational activities, as well as difficulties in paying utilities. Moreover, the survivors used their savings, borrowed money, or did not pay their credit card bills, which were included in FO. For example, the item ‘I had to use my savings’ quantifies the financial strain faced by the COVID-19 survivors. It designates whether the survivors are required to consume their reserved funds to manage the outlays due to the disease, e.g., survivors might have used savings to pay for medical bills not covered by insurance, cover lost income during quarantine, or cope with the additional expenses of medications and home care.

**Financial hindrance (FH):** The FH encompasses the four items of COVID-19 financial burden that forced survivors to skip treatment due to the expense of medicine, transportation, groceries, and utility bills. The difference between FO and FH is that in FO, survivors either use their savings or cut their budget to cope with the financial burden. In contrast, the survivors were unable to meet the expenses incurred in FH due to COVID-19 infection. The statement of the scale ‘I skipped my treatment just because of the expensive medicine,’ reveals the economic barriers COVID-19 survivors might experience in retrieving essential healthcare. For example, a survivor might have been prescribed antiviral drugs or post-COVID recovery medications but did not purchase them at high cost. Another example could involve someone avoiding physiotherapy or follow-up visits due to the unaffordability of the associated expenses.

**Hostile behavior (HB):** The HB scale used four items to measure the survivor’s antagonistic behavior encountered during the infection period. These items include threats, insults, screams, physical assaults, or curses from family members, lenders, or others for not copying with the financial responsibilities due to COVID-19 infection and the elevated financial burden.

**Care and lifestyle (CC):** This construct was measured through three items designed to quantify the impact of COVID-19 on survivors’ finances regarding their care, belongings, and products used in their daily routine, which define their lifestyle, such as a car, house, jewelry, mobile, etc.

**Seeking support (SS):** The SS construct comprises four items designed to quantify the financial stress experienced during the COVID-19 infection. These items reflect the survivor’s dependence on others to cope with the economic impact due to infection.

**Psychosocial affects (PA):** This construct was based on five items to depict the impact of the financial burden on survivor’s emotional, psychological, and social spectrum in a broader sense.

**Economic burden (EB):** The latent variable EB was measured through a twenty-seven-item items scale representing the COVID-19 survivor’s financial hardships during the disease period.

**Fig 1:**
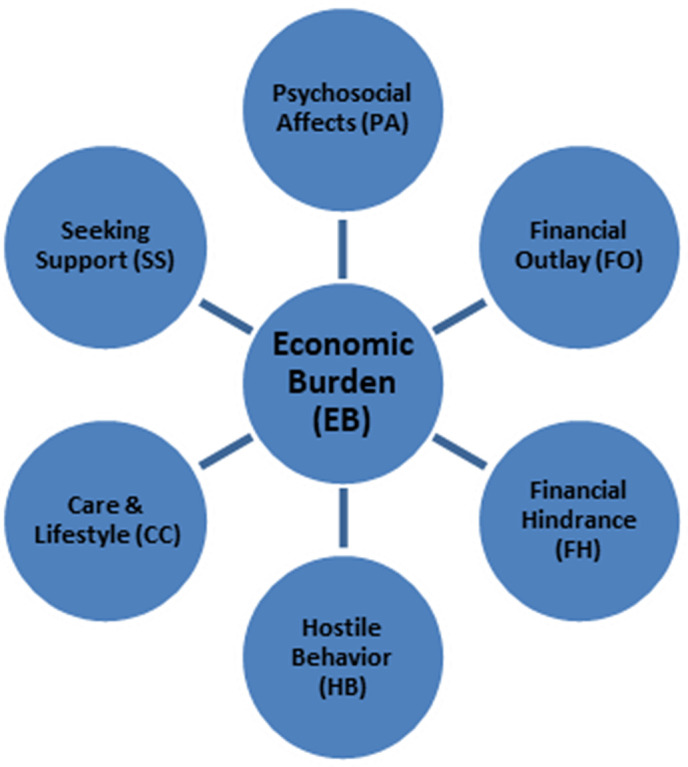
Proposed research model for the EB and the associated six constructs.

The proposed model for estimating the financial burden of COVID-19 survivors, based on six constructs, is illustrated in Fig 1.

**Fig 2:**
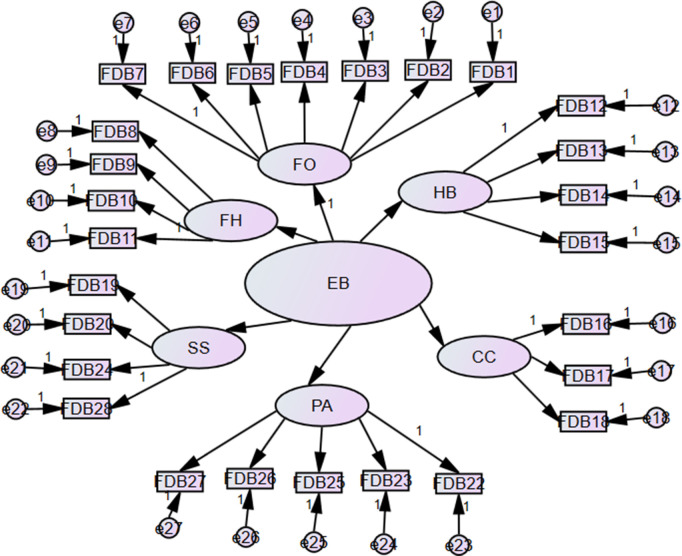
Proposed latent variable EB model based on the six constructs and the associated item.

Since the latent variable EB is estimated based on six constructs with varying numbers of items, a structural equation model (SEM) [21] will be employed to estimate EB. The SEM for the latent variable EB, using the six constructs and their associated items, is presented in Fig 2.

The path model specified in Fig 2 is defined in SEM notation as:


$${𝐗}_{𝐢}=\Lambda_{𝐗}\xi_{𝐤}+\theta_{\delta }$$ (1)

where ${𝐗}_{𝐢}$ is a column vector of items used to measure the constructs, $\xi_{𝐤}$ is a column vector of constructs, $\Lambda_{𝐗}$ is a matrix of coefficients of relationships between the items and the constructs, and $\theta_{\delta }$ is a variance-covariance matrix of relations among the residual terms of exogenous variables (𝐗).

Equation (1) depicts that each construct is characterized by a structural equation relating $\Lambda_{𝐗}$, $\xi_{𝐤}$, $\theta_{\delta }$. This means that a set of items is used to define the constructs intended to measure as well as the residual, which is a variation specific to the variable unrelated to the construct. To estimate the model specified in (1), we incorporate a k×k variance-covariance matrix Φ of relationships among the constructs $\xi_{𝐤}$. The Equation (1) takes the form as:


$$\Sigma \left(\hat{\theta }\right)=\Lambda_{𝐗}\Phi \Lambda_{𝐗}+\theta_{\delta }$$ (2)

The maximum likelihood estimates of (2) are obtained by using the likelihood function [21]:


$$F_{MLE}\left(\theta \right)=\log\left|\Sigma \left(\theta \right)\right|+tr\left(𝐒\Sigma {\left(\theta \right)}^{-1}\right)-\log\left|𝐒\right|-p$$ (3)

where, 𝐒 denotes sample covariance matrix and p represents the number of items.

There is currently no universally accepted measure to assess overall model fit, so we must rely on multiple fit criteria. These indices serve to evaluate how well a model fits the data. In structural equation modeling (SEM), a range of goodness-of-fit indices is available for evaluating model fit. The model specified in (2) is assessed through common goodness-of-fit indices. These include:

**Goodness-of-Fit Index (GFI):** The GFI quantifies the proportion of variance-covariance of 𝐒 that is predicted by Σ(θ). This index is an equivalent of $R^{2}$ in regression analysis and is defined as:


$$GFI=1-\frac{tr\left[{\left(\Sigma^{-1}S-I\right)}^{2}\right]}{tr\left[{\left(\Sigma^{-1}𝐒\right)}^{2}\right]}$$ (4)

The range of GFI is from 0 to 1, and the least acceptable value for a model is at least 0.7 [22].

**Adjusted Goodness of Fit Index (AGFI):** The GFI is sensitive to sample size and is influenced by the number of parameters. AGFI is a modified version of GFI, adjusted to accommodate a different number of parameters. This index is again an analog of adjusted $R^{2}$ as in regression analysis and is defined as:


$$AGFI=1-\left[\frac{k\left(k+1\right)}{2df}\mathrm{\backslash rightleft}(1-GFI\right)$$ (5)

The AGFI ranges from 0 to 1, and this index also has an acceptable model range of 0.7 or more [23].

**Chi-square/degrees of freedom (**χ2/χ2df\nulldelimiterspacedf**):** This statistic assesses the likelihood of the difference between the population covariance matrix Σ and the sample covariance matrix 𝐒. It is defined as:


$$\chi^{2}/df=\left\{\left(N-1\right)F\left(\theta \right)\right\}/df$$ (6)

and, df=k(k+1)/2−t

where, t is the number of parameters to be estimated. However, the acceptable range for this statistic is less than three. The statistic is highly sensitive to sample size and is not a good measure for large sample sizes [24].

**Root mean square error (RMSE):** The RMSE quantifies model adequacy by considering both the model’s complexity, in terms of the number of parameters estimated, and the model’s discrepancy. It is defined as:


$$RMSE=\sqrt{\frac{\chi^{2}-df}{\left(N-1\right)}}$$ (7)

The statistic ranges from 0 to 1, and the least acceptable value for model adequacy is 0.08 [25].

**Root mean square error approximation (RMSEA):** The RMSEA is a modified version of RMSE defined in Equation (7). It is adjusted for the model degrees of freedom and defined as:


$$RMSEA=\sqrt{\frac{\chi^{2}-df}{df\left(N-1\right)}}$$ (8)

The RMSEA ranges from 0 to 1, and the least acceptable value for accepting a fitted model is 0.08 [25]. The data were entered, screened, and analyzed using SPSS v26, AMOS v24, and the R language.

## 3. Results

This section will provide the descriptive and analytical results of the study. Out of 5045 survivors, 59.2% were male, 23.4% were 25 years old, 35.1% were 26–50 years old, 41.6% were above 50 years old, 63.5% were married, 65.8% were employed in some job, and 64.6% had some college or university level education. About 29.8% of the COVID-19 survivors were admitted to hospital, out of which 15.9% were in ICU, 4.3% faced permanent disability, 72.5% had mild to moderate infection, and 76.8% of the survivors disease duration was less than 15 days. The average age of the survivors was 45.4 years, the duration of the disease was 12.1 days, the daily cost was 4763 PKR, the morbidity cost was 21286 PKR, and the number of morbidity days was 12 days.

**Fig 3:**
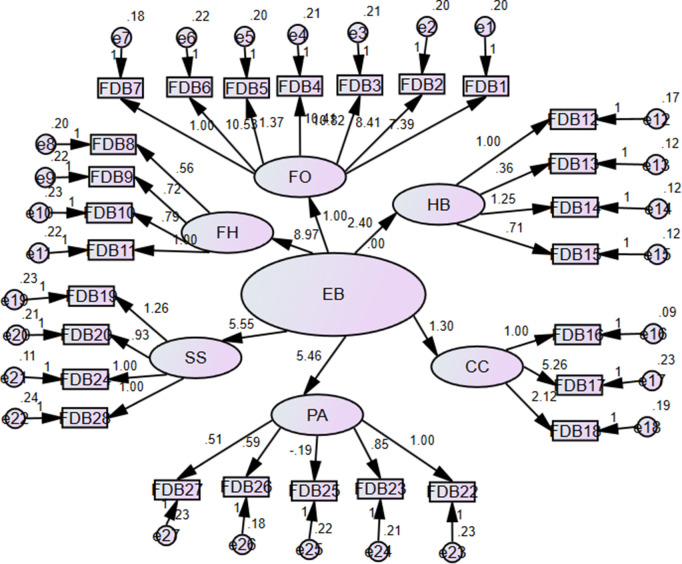
Estimated unstandardized coefficients for the survivor's financial burden model.

**Fig 4:**
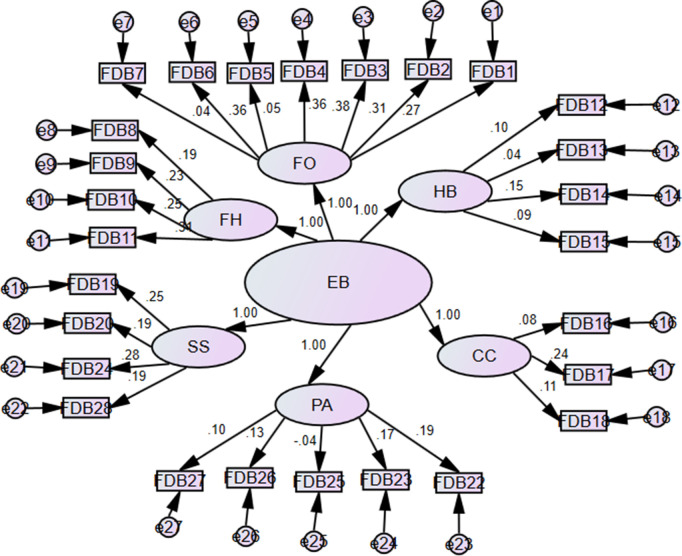
Estimated standardized coefficients for the survivors financial burden model.

The Economic Burden (EB) scale was developed based on six constructs: financial outlay (FO), financial hindrance (FH), hostile behavior (HB), care and lifestyle (CC), seeking support (SS), and psychosocial affect (PA). These constructs were confirmed through confirmatory factor analysis (CFA) [26]. The estimated proposed path model, with unstandardized coefficients, is presented in Fig 3, and the standardized coefficients are shown in Fig 4.

The unstandardized and standardized regression weights, along with their respective error variances, for the estimated EB model are presented in Table 2.

**Table 2:** Unstandardized and standardized regression coefficients with respective error variances for the estimated EB model.

Items	Weights		Error Variances
	Unstandardized	Standardized	
FDB1: I had to use savings.	7.386^**^	0.271	0.205^***^
FDB2: I had to borrow money or take out a loan.	8.412^**^	0.310	0.197^***^
FDB3: I could not make payments on credit cards or other bills.	10.822^**^	0.381	0.205^***^
FDB4: I cut down on spending for food and/or clothes.	10.409^**^	0.362	0.214^***^
FDB5: I cut down on spending for health care for other family members.	1.368^*^	0.053	0.199^***^
FDB6: I cut down on recreational activities.	10.528^**^	0.363	0.217^***^
FDB7: I cut down on expenses in general.	1.000	0.040	0.181^***^
FDB8: I skipped my treatment just because of the expensive medicine.	0.556^***^	0.187	0.203^***^
FDB9: During covid-19, I avoided myself to going hospital just because of transportation fare.	0.721^***^	0.231	0.220^***^
FDB10: During the disease period, I was worried that the grocery I bought just didn’t last and I didn’t/do have money to get more.	0.793^***^	0.247	0.231^***^
FDB11: For not giving proper time to my job/work the electric, gas, oil, or water (utility) company threatened to shut off services in my home?	1.000	0.311	0.223^***^
FDB12: During that time someone, including family, threaten me with harm for not meeting the financial responsibilities.	1.000	0.101	0.166^***^
FDB13: Does anyone from which you have taken debt threaten you for not giving back the debt?	0.358^**^	0.043	0.117^***^
FDB14: Did anyone, including family, insult/ scream/ curse or talk down to you for not handling the family life/ meeting the financial responsibility properly?	1.251^***^	0.149	0.119^***^
FDB15: Did anyone, including family, physically hurt you for not handling the family life/ meeting the financial responsibilities properly?	0.711^***^	0.086	0.117^***^
FDB16: Covid-19 kept me away from taking a health check on myself from regular doctor appointments.	1.000	0.076	0.087^***^
FDB17: I delayed or avoided my personal treatment, follow up or a recommended procedure due to financial concerns?	5.264^***^	0.238	0.232^***^
FDB18: I sell some of my belongings (house, car, jewelry, mobile, any valuable thing) for the treatment of Covid-19/ coping with financial problems.	2.123^***^	0.108	0.191^***^
FDB19: During the disease period, I depend on someone else for income or self-sufficient?	1.263^***^	0.246	0.227^***^
FDB20: I financially support some other Covid-19 affectee, to help them meet their treatment/household expenses.	0.932^***^	0.190	0.213^***^
FDB22: I fear/experienced a loss of income/ employment/ work as a result of my time diversion towards Covid-19 treatment.	1.000	0.194	0.108^***^
FDB23: I had faced difficulties in paying for daily household expenses then.	0.850^***^	0.172	0.239^***^
FDB24: I am currently in debt as a result of treatment expenses.	1.005^***^	0.281	0.225^***^
FDB25: My financial condition was satisfactory to me then.	−0.188^*^	−0.037	0.210^***^
FDB26: I or my family experienced financial hardships as a result of the Covid-19.	0.595^***^	0.130	0.223^***^
FDB27: My financial condition worsened as a result of Covid-19.	0.507^***^	0.099	0.182^***^
FDB28: I discussed my health/ personal/ financial issues with anyone during that time.	1.000	0.192	0.231^***^

* p < 0.10, ** p < 0.05, *** p < 0.01.

The questionnaire items are significantly associated with their respective constructs and the latent variable EB (p < 0.05). The standardized regression coefficient of 10.53 shows that the statement ‘I cut down on recreational activities’ has a significant (p < 0.05) impact on the financial outlay domain and, consequently, on the financial burden. This statistic indicates the strength of the association between the item and the latent variable of financial outlay. The standardized regression coefficient of 0.363 further shows that this item has a moderately strong effect on the financial outlay domain. In turn, the financial outlay domain contributes to the overall financial burden. Hence, decreasing recreational activities plays a significant role in the modulation of the financial burden felt by COVID-19 survivors, as reflected in the financing part of the theoretical model. On a similar note, the other items can be described in SEM results presented in Table 2.

The survivors reported that they used their savings and even borrowed money to cope with their financial needs due to COVID-19 treatment. They were unable to pay the credit card bills. COVID-19 survivors have been deeply affected in their spending habits, with shifts in priorities, supply disruptions, and economic challenges influencing their food and clothing expenditure patterns. The economic burden of the disease restricts their ability to spend on health concerns for other family members. The survivors adjusted their recreational habits to prioritize health, manage anxiety, and adapt to the challenges posed by the pandemic. The financial burden of medical treatment overwhelmed the survivors and forced them to skip treatment due to expensive medicines or transportation fares. Food insecurity was another concern for the survivors, in addition to the nonpayment of utility bills. Some of the survivors reported that they experienced hostile behavior during the pandemic period. The survivors sold their personal belongings, which held sentimental value, as a desperate measure for survival. The COVID-19 survivors have faced worsened financial conditions due to medical expenses, lost income, long-term health effects, and increased stress.

The proposed survivor’s EB model was assessed for acceptance or rejection using multiple goodness-of-fit indices. These indices for the estimated model are provided in Table 3.

**Table 3:** The goodness of fit indices of the proposed EB SEM model.

Indices	Values
GFI	0.931
AGFI	0.920
Chi-square/ df	14.530
RMR	0.009
RMSEA	0.053

The chi-square/df goodness of fit index is greater than the acceptable cutoff ($\chi^{\text{2}}/df<\;\text{5}$) and is a poor index to use for sample sizes over 200 [27]. The goodness of fit indices less or not sensitive to sample sizes reported in Table 1 are in an acceptable range with respect to their cutoffs, e.g., GFI=0.931 (>0.90), AGFI=0.920 (>0.90), RMR = 0.009 (<0.05) and RMSEA = 0.053 (<0.08) [28].

The results presented in Table 4 are the univariate odds ratios (ORs) with 95% confidence intervals for COVID-19 patient profile variables and the six constructs of the EB.

**Table 4:** Odds ratio with 95% CI of six EB constructs with survivors profile.

Profile	Categories	Financial Outlay	Financial Hindrance	Hostile Behavior	Care and Lifestyle	Seeking Support	Psychosocial Affect	Economic Burden (Overall)
**Gender**	Male							
	**Female**	**1.162** [0.892, 1.513]	**0.949** [0.763, 1.180]	**0.963** [0.720, 1.290]	**1.035** [0.811, 1.322]	0.747** [0.597, 0.935]	1.272* [0.967, 1.672]	**1.012** [0.633, 1.62]
**Age**	≤ 45 years							
	**> 45 years**	1.388** [1.064, 1.811]	1.219* [0.979, 1.518]	**1.225** [0.915, 1.641]	1.316** [1.031, 1.681]	**1.139** [0.909, 1.426]	**1.125** [0.858, 1.476]	2.153*** [1.338, 3.465]
**Marital Status**	Single							
	**Married**	1.558*** [1.189, 2.041]	**1.145** [0.917, 1.429]	0.714** [0.532, 0.959]	0.637*** [0.497, 0.818]	**1.181** [0.940, 1.484]	1.314* [0.988, 1.721]	**1.488** [0.923, 2.398]
**Occupation**	Employed							
	**Unemploye**d	**1.125** [0.856, 1.478]	**1.046** [0.835, 1.311]	**0.960** [0.524, 1.299]	**1.019** [0.791, 1.313]	**0.996** [0.790, 1.256]	**0.853** [0.643, 1.132]	**1.025** [0.631, 1.667]
**Education**	Above Matriculation							
	**Up to Matriculation**	1.287* [0.981, 1.686]	1.393*** [1.114, 1.742]	**1.054** [0.781, 1.422]	1.464*** [1.139, 1.880]	1.259** [1.001, 1.585]	**1.075** [0.812, 1.424]	2.183*** [1.348, 3.534]
**Hospital Admission**	No							
	**Yes**	**1.066** [0.803, 1.414]	1.441*** [1.140, 1.821]	1.495** [1.095, 2.037]	1.319** [1.014, 1.715]	1.318** [1.036, 1.675]	**1.201** [0.895, 1.610]	2.320*** [1.402, 3.846]
**Disease Duration**	≤ 14 days							
	**> 14 days**	1.543*** [1.133, 2.101]	0.497*** [0.383, 0.644]	0.153*** [0.103, 0.226]	0.236*** [0.175, 0.320]	0.454*** [0.347, 0.595]	1.561*** [1.131, 2.155]	0.198*** [0.113, 0.347]
**ICU**	No							
	**Yes**	**1.122** [0.580, 2.174]	**1.228** [0.712, 2.123]	**1.513** [0.756, 3.030]	**1.359** [0.744, 2.481]	**0.816** [0.462, 1.441]	**0.870** [0.426, 1.776]	**1.570** [0.457, 5.376]
**Infection**	Mild/Moderate							
	**Severe**/Critical	1.340** [1.004, 1.789]	**1.035** [0.815, 1.315]	**0.942** [0.684, 1.299]	**1.088** [0.832, 1.423]	1.360** [1.065, 1.738]	1.531*** [1.134, 2.068]	2.114*** [1.264, 3.537]
**Permanent Disability**	No							
	**Yes**	**0.762** [0.402, 2.481]	**1.250** [0.740, 2.110]	2.959*** [1.558, 5.618]	1.686* [0.942, 3.021]	**0.825** [0.480, 1.418]	**0.890** [0.462, 1.715]	**1.548** [0.498, 4.762]

* p < 0.10, ** p < 0.05, *** p < 0.01.

The OR of 1.162 for females indicated that females were 1.162 times more likely to be frugal than male survivors due to COVID-19 infection. This means females faced higher budget constraints than male survivors in the study population. The odds ratio (OR) of 1.388 for the age variable indicates that older survivors had 1.388 times the frequency of occurrence (FO) compared to survivors aged 45 years or younger. This means that as the age of survivors increases, their financial outlays increase by a factor of 1.388, holding other variables constant. The odds ratio (OR) of 1.125 for survivors’ occupation showed that unemployed survivors had 1.125 times greater budget constraints than employed survivors. The odds ratio (OR) of 1.125 for survivors’ occupation indicates that unemployed survivors had 1.125 times higher odds of experiencing budget constraints compared to employed survivors. This means that being unemployed is associated with a slightly increased likelihood of facing financial limitations relative to being employed, when other factors are held constant. The survivors with education levels up to matric faced 1.287 times higher cuts in their routine budgets than those with post-matriculation education. Education and the survivor’s financial budget management skills are positively associated. A survivor with a higher education tends to manage the disease’s progression well. The survivors admitted to the hospital faced comparatively 1.066 times higher FO than the survivors with no hospitalization due to the COVID-19 infection. The survivors with a disease duration of more than 14 days reported 1.543 times the elevated FO than the survivors with a disease duration of 14 days or less. The survivors admitted to the hospitalized survivors faced 1.122 times more FO than the non-ICU admitted survivors. The survivors with severe or critical conditions bared 1.34 times more FO than those with mild or moderate infection conditions. The survivors exposed to some permanent disability due to COVID-19 infection reported less FO (OR=0.762) than the survivors with no permanent disability.

The odds ratio (OR) of 0.949 for the variable gender indicated that female survivors experienced fewer fractures (FH) than males. The survivors with more than 45 years of age reported 1.219 times higher inability to cope with the disease financial burden than the survivors with 45 or less years of age. The married survivors practised 1.145 times more elevated FH than the single survivors. The unemployed survivors faced slightly 1.046 times more financial hardship (FH) than the employed survivors. Survivors with education at matriculation or below level confronted 1.393 times higher FH than the rest. The OR of 1.441 for the variable hospitalization depicted that the survivors with exposure to hospitalization experienced 1.441 times more FH than those not admitted to the hospital for COVID-19 treatment. Among the hospitalized survivors, the ICU survivors reported 1.228 times higher FH than the non-ICU survivors. The survivors with severe or critical conditions revealed 1.035 times greater FH than the survivors with mild or moderate conditions of infection. The survivors exposed to some permanent disability due to COVID-19 infection reported earlier faced less FO (OR=0.762) but higher FH (OR=1.250) than the survivors with no permanent disability.

The male survivors encountered slightly higher HB (OR=1.038) than the females. The survivors with age more than 45 years faced 1.225 times greater HB than the survivors with 45 or less years of age. The married, unemployed, more than 14 days of disease duration, and severe or critical conditioned survivors experienced fewer times of HB than the competitors. Survivors with up to a matriculation level of education were 1.054 times more exposed to HB than those with higher secondary education. The survivors admitted to the hospital or ICU or faced some permanent disability experienced an elevated HB situation than the antagonists.

The female survivors reported that their care and lifestyle (CC) were comparatively more affected (OR=1.035) than the male survivors. The elderly survivors (≥ 45 years) reported their CC as more devastated (OR = 1.316) than the younger survivors (< 45 years). The single survivors were more distressed (OR=1.570) than the married survivors regarding their well-being practices and daily living. The CC had a comparatively elevated effect on unemployed survivors (OR = 1.019) compared to the employed. Once again, education had an adverse effect on survivors self-care and daily habits. The survivors with less education were more likely to be at risk (OR=1.464) concerning health maintenance and lifestyle than the more educated survivors. Self-care and daily habits were more affected among hospitalized (OR = 1.319) and ICU (OR = 1.359) survivors than among non-hospitalized or non-ICU survivors. The survivors with severe or critical infection had 1.088 times higher devastated CC than the survivors with mild or moderate levels of infection. The survivors who faced some permanent disability in response to COVID-19 infection experienced 1.686 times higher affected wellness and living than the opponents.

The female survivors sought less (OR=0.747) financial support than the males. The survivors aged more than 45 sought comparatively more financial support than those aged 45 years or younger. The married survivors requested more financial aid (OR=1.181) than the singles. The comparatively less educated survivors explored more monetary assistance (OR=1.464) than the educated survivors. The survivors admitted to the hospital (OR=1.318) sought more financial aid than the non-hospitalized survivors. The COVID-19 survivors with a disease duration of 14 days or less had sought more financial support (OR=4.237) than those with a longer disease duration.

The emotional and social impacts were more adversely prevalent among female survivors (OR=1.272) than the males. Elderly survivors reported elevated behavioral and emotional outcomes (OR=1.125) than the younger survivors. The married survivors experienced 1.314 times greater psychological and relational impacts than the singles. The odds ratio (OR) of 1.172 for the variable occupation revealed that employed survivors had 1.172 times the PA of the unemployed. Survivors with education up to matriculation had psychosocial effects 1.075 times higher than those with higher secondary education. The hospitalized survivors practiced 1,201 times more physical activity (PA) than the non-hospitalized survivors. The longer disease duration survivors had 1.561 times greater mental and interpersonal repercussions than the survivors with a disease duration of up to 14 days. The non-ICU survivors reported less PA than the ICU survivors (OR=0.870). The survivors with severe or critical infection status had 1.531 times higher emotional and societal implications than the survivors with mild or moderate symptoms of infection.

**Fig 5:**
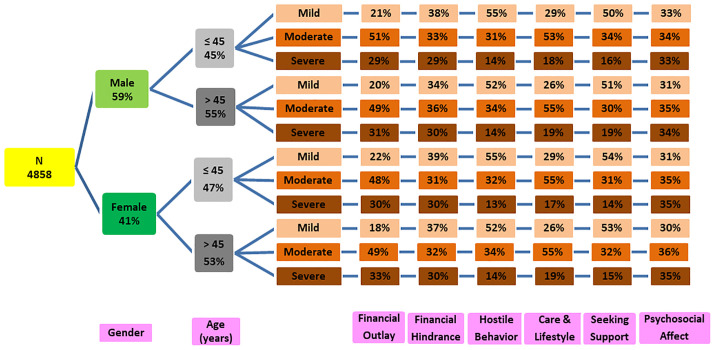
Gender- and Age-wise Tree Diagram of Financial Burden of Survivors with Respect to Constructs.

**Fig 6:**
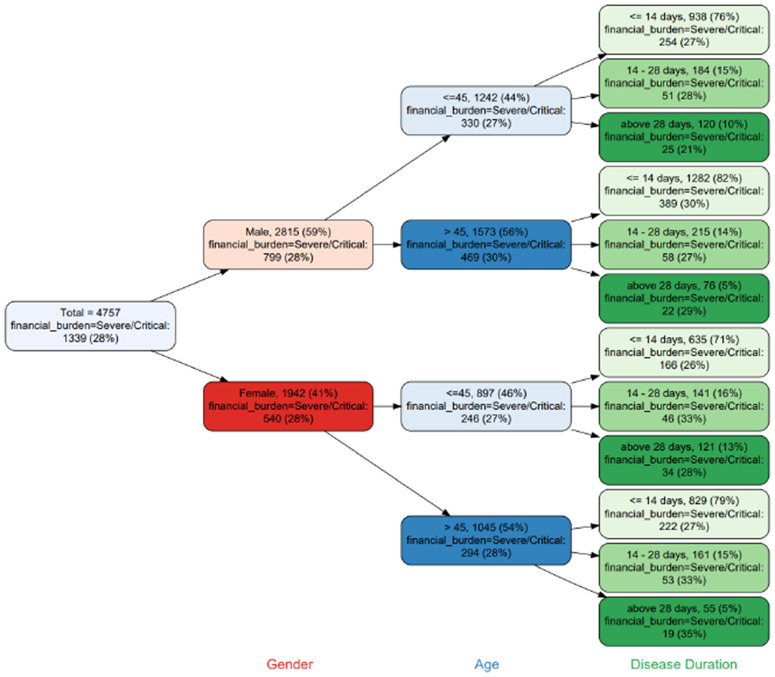
Tree diagram of Survivors with severe/critical EB with respect to gender, age and disease duration.

The financial strain among the female survivors (OR=1.012) was comparatively higher than the male survivors. The survivors older than 45 years had 2.153 times the economic impact of survivors aged 45 years or less. The married survivors faced 1.488 times more financial load than the singles. The unemployed survivors portrayed comparatively 1.025 times higher financial stress than the employed survivors. Survivors with education up to matriculation reported 2.183 times greater financial encumbrance than those with postsecondary education. The hospitalized survivors experienced 2.32 times more financial stress than the non-hospitalized survivors. Survivors with a disease duration of up to 14 days faced a greater financial burden than those with a disease duration of more than 14 days (OR = 5.051). The ICU survivors reported a 1.570 times higher monetary burden than the non-ICU survivors. The severe or critical diseased survivors had 2.114 times raised EB than the survivors with mild or moderate financial strain. The survivors with permanent disability as a result of the infection experienced 1.548 times higher fiscal hardships than the competitor group. Similar findings are observed from Figs 5 and 6 where the economic burden of the survivors are categorized as mild, moderate and severe, and classified with respect to gender and age.

## 4. Discussions

The present study quantifies the financial burden of COVID-19 on survivors in Punjab, Pakistan, as also explored in the USA [29], the United Kingdom [29], India [30,31], Iran [31,32], Saudi Arabia [33], and globally [34]. The study results revealed that female survivors suffer more economically than male survivors (OR = 1.012). This means female survivors experienced greater economic challenges than male survivors. The potential reasons could be that females are more likely to work in sectors heavily impacted by pandemic-related closures or reduced economic activity, such as hospitality, retail, and healthcare. This can lead to job loss, reduced working hours, or income instability. The findings coincide with the [7]. The survivors over 45 years had more financial hardships during COVID-19 than their younger counterparts, as found in [7,35,36]. This inequality resulted from several factors, including unstable employment rising healthcare expenses, few opportunities for reemployment and growing financial obligations. The findings of the study revealed that married survivors are greatly affected by the pandemic than the single survivors and are supported by [37–43]. The results disclose the impact of marital status on a number of aspects of survivors experienced during the diseased period. Higher level of stress and anxiety might resulted from married survivors having more domestic duties such as managing family dynamics and providing care. Financial stress in married survivors can also be made worse by economic pressures like shared financial responsibilities and possible job losses. Therefore, the married survivors faced an increased impact from the COVID-19 due to the emotional, financial and social ramifications of marriage.

The research results revealed that survivors with postsecondary education faced less financial strain than those with secondary or lower education. This finding is also supported by [13,44–47]. These studies suggest that a higher education level can help protect against economic shocks, as it is often associated with increased employability, job stability, and higher earning potential. Those with postsecondary education are more likely to work remotely in their respective profession, which reduces the risk of job loss or salary deductions. On the other hand, secondary or lower education survivors might work in industries such as retail or hospitality, which have been severely affected by lockdowns and restrictions and are more susceptible to economic disruptions. Higher education is strongly associated with greater literacy and access to tools that resolve financial difficulties during pandemics. In many studies, including our findings, hospitalized survivors faced a higher financial burden [48–51].

The financial expenditure of COVID-19 patients is one of the long-term effects on the development of the health sector’s financing and budgeting due to the pandemic’s significant impact on hospitals’ operational, economic, and financial dimensions. The pandemic caused a direct reduction in public hospitals’ revenue levels due to the higher costs and a significant decrease in incomes as the Pandemic emergency caused most patients to avoid or postpone treatment for infection and non-infection diseases. Financial hindrance of COVID-19 patients is a global concern since the pandemic has increased the financial constraint to individuals by a significant margin and over a considerable geographical scale. The expenditures involved in seeking medical attention, including hospitalization, medication, and other healthcare interventions, are high, placing a significant burden on patients and their households. Moreover, many have lost their sources of income or had their income substantially reduced during the pandemic era. Consequently, financial hindrance has been a significant constraint for COVID-19 patients. The pandemic requires a comprehensive policy response, ranging from affordable health policy interventions to income stability assistance and reducing socioeconomic disparities in response to COVID-19, whether or not individuals are suffering from the pandemic.

Hostile behavior, a complex problem determined by fear, anxiety, and a lack of control in COVID-19 sufferers, can be managed or minimized by healthcare professionals by thoroughly understanding the underlying causes and forms of hostile behavior during the epidemic. Experts also found that controlling angry behavior and maintaining a consistently polite and safe hospital environment during the pandemic might require empathy, open communication, and psychological assistance. Many COVID-19 patients have been negatively impacted by disruptions to standard medical care, including postponed preventive screenings, elective surgeries, and long-term management of comorbid conditions, as overwhelmed healthcare systems struggle to manage the pandemic and fear of viral spread keep patients away from healthcare settings. Due to the importance of reducing virus spread, many COVID-19 patients must adjust their daily behaviors, including following isolation guidelines, maintaining rigorous personal hygiene, and changing work practices and social activities to avoid inadvertently spreading the infection. Due to the COVID-19 pandemic, many survivors have altered their lifestyle habits to become healthier, such as maintaining good hand hygiene, wearing masks in public settings, and being more mindful of their personal health.

COVID-19 patients with financial challenges often find other sources of peer support and financial assistance from various organizations and programs. Such programs offset patients’ non-medical expenses, such as rent, mortgage, utilities, and food, playing a critical role in providing the needed support to those affected. The pandemic has significant psychosocial effects on patients, including its influence on their mental wellness, emotional health, and social relationships. Most patients are anxious about their health outcomes and that of the pandemic, increasing their stress levels over their future. Patients in the general population have reported stress and anxiety related to the transmission and expansion of the virus in their communities. Depression levels have also increased, among other mental health conditions such as PTSD. The pandemic has resulted in significant losses, including income loss, job loss, and bereavement for others, causing grief and mourning among the affected. COVID-19 patients, especially those in isolation and who have been hospitalized, also experience loneliness and a desire for social connection. In addition, some of the patients reported stigma from their communities, and others avoided associating with them for fear of contracting the virus. Patients also reached out to their friends, families, and support systems for social connections and emotional support. They also practiced wellness activities, such as mindfulness and meditation, to support their mental well-being. The present study has some limitations, including the reliance on self-reported data, which may introduce a recall bias. Another potential limitation could be the cross-sectional design, which may restrict causal inference.

## 5. Conclusion

The study reveals that the long-term economic effects of COVID-19 on survival in Punjab, Pakistan, pose a significant challenge, particularly among vulnerable groups, including older individuals, women, and those who are unemployed. To overcome these challenges, the following policies are proposed. First, Pakistan needs to implement a policy of affordable/ free treatment for Covid 19 survivors like it has been adopted in South Korea and Germany, where the state takes the primary responsibility for payment of health bills. This may help to decrease out-of-pocket costs and relieve financial burdens. Second, wage-loss programs, such as cash payments for survivors who receive little or no wages due to sickness, should be extended, similar to stimulus and unemployment benefit packages, as seen in the United States and the United Kingdom. Such measures would ensure that survivors did not spend all their money or have no money to cover their basic needs after the disasters. Third, debt relief and loan restructuring programs can be adopted for individuals who took loans for medical treatment, as seen in the Indian context. Possible securities for recovering the vulnerable through microfinancing schemes could be added. Lastly, the establishment of corresponding social protection policies toward the social protection needs of target groups such as women and the unemployed or differently abled, like the Bolsa Família for the needy families in Brazil that have provided the families social insurance cover for health. Such extensive strategies would significantly reduce the post-COVID-19 economic and social impact on Pakistan survivors. In addition, establishing a national registry to monitor the long-term outcomes can guide resource allocation and strengthen support systems for COVID-19 survivors in Pakistan. The study is extendable to other provinces of the country for more generalizations. Longitudinal studies can also be helpful in assessing the sustained financial challenges that survivors face.

## Supporting information

S1 File SM–COVID-19 survivors data. (XLSX)
